# Effect of fibrin on the expression of adhesion molecules (ICAM-1, ITGAV, and ITGB3) in unrestricted somatic stem cells

**DOI:** 10.1016/j.htct.2025.103827

**Published:** 2025-05-02

**Authors:** Sanaz Khaseb, Mahdi Kohansal Vajari, Mina Soufi Zomorrod, Maryam Rezai Rad, Monireh Ajami, Mansoureh Ajami, Saba Sadeghpour, Amir Atashi

**Affiliations:** a Faculty of Medical Sciences, Tarbiat Modares University (TMU), Tehran, Iran; b School of Allied Medical Sciences, Kerman University of Medical Sciences, Kerman, Iran; c Research Institute for Dental Sciences, Dental Research Center, School of Dentistry, Shahid Beheshti University of Medical Sciences, Tehran, Iran; d Faculty of Paramedical Sciences, Tehran Medical Sciences Branch, Islamic Azad University, Tehran, Iran; e School of Allied Medical Sciences, Shahroud University of Medical Sciences, Shahroud, Iran; f School of Medicine, Shahid Beheshti University of Medical Sciences, Tehran, Iran; g Stem Cell and Tissue Engineering Research Center, Shahroud University of Medical Sciences, Shahroud, Iran

**Keywords:** Adhesion molecule, Expansion, Fibrin, Umbilical cord blood, Unrestricted somatic stromal cells

## Abstract

**Background:**

Hematopoietic stem cell expansion relies on direct cell-cell interactions mediated by adhesion molecules, integrins, and cytokines. Unrestricted somatic stem cells have emerged as novel stromal cells supporting hematopoietic stem cell expansion in co-culture conditions via secretion of hematopoiesis-related cytokines and the expression of adhesion molecules. Previous research showed fibrin increased hematopoiesis-related gene expression in these cells. This study focused on the adhesive characteristics of unrestricted somatic stem cells on 3D fibrin scaffolds.

**Methods:**

Unrestricted somatic stem cells were isolated from umbilical cord blood and characterized using flow cytometry and multilineage differentiation assays. Scanning electron microscopy and DAPI staining were employed to analyze cell attachment to fibrin. Viability on fibrin was assessed through MTT assays. Quantitative polymerase chain reaction was conducted to evaluate the expression of intercellular adhesion molecule 1 (ICAM-1), integrin subunit αv (ITGAV), and integrin subunit β3 (ITGB3) in cells cultured on 3D fibrin scaffolds.

**Results:**

Cells were positive for CD73, CD105, and CD166 but negative for CD45. Alizarin red and Oil red O stains confirmed calcium deposition and lipid vacuoles. MTT assays revealed that fibrin positively impacts viability. ITGAV expression was significantly increased in cells cultured on fibrin compared to those cultured on plastic tissue culture plates (Control Group). Furthermore, ITGB3 expression showed no significant change in both groups, while ICAM-1 expression was downregulated in cells cultured on fibrin.

**Conclusions:**

Our study revealed that fibrin has a positive impact on the expression of ITGAV, which plays a crucial role in direct cell-cell interactions affecting hematopoietic stem cell expansion.

## Introduction

According to the European Society for Blood and Marrow Transplantation, hematopoietic stem cell transplantation (HSCT) is a potentially curative therapy for several life-threatening diseases including, solid tumors, immune disorders and hematological malignancies with acute myeloid leukemia being the most frequent indication for allogeneic HSCT, followed by acute lymphoblastic leukemia in Europe.^1^ Although an HLA-matched sibling is the preferred donor, only approximately 30% of patients who could benefit from HSCT have such a donor available. One of the available options for tackling this issue is the manipulation of umbilical cord blood (UCB) -hematopoietic stem cells (HSCs) due to less stringent requirements for HLA matching.^2^ However, despite all the advantageous aspects of UCB, its primary drawback is the low yield of HSCs in comparison to bone marrow (BM) or peripheral blood-mobilized HSCs. Consequently, this leads to complications including delayed hematological recovery, higher graft failure rates, and risk of infection.^3^

Great efforts have been dedicated to overcoming this limitation by expanding the number of HSCs both *in vivo* and *in vitro*. One of the applied methods is co-culture protocols developed for the expansion of UCB-HSC.^4^ Mesenchymal stem cells (MSC), one of the cord blood cells used in co-culture, functions as a support for HSCs.^5^ Different studies demonstrate that MSCs in NOD/SCID mice induce engraftment of UCB-derived CD34^+^ cells.^6^^,^^7^ In addition to MSCs, another UCB-derived cell termed unrestricted somatic stem cell (USSC) can also promote the expansion of HSCs.^8^ This rare CD45-negative population grows adherently and can be expanded to 10^15^ cells without losing pluripotency.^9^ Hashemi et al. used MSCs and USSCs as feeder layers to increase the population of UCB-CD34^+^ cells for bone marrow transplantation.^10^ Another study also reported that USSCs significantly supported the proliferation of HSCs in the bone marrow of NOD/SCID mice and showed no sign of tumorigenicity.^11^ The possible underlying reason for the positive influence of USSCs on HSC proliferation is the production of hematopoiesis-supporting cytokines. Compared to MSCs, these cells produce significantly more hematopoiesis-related cytokines such as stem cell factor (SCF) thus making them a better candidate for stroma-driven *in vitro* expansion of UCB-HSCs.^8^

It is worth noting that the interaction of the HSCs with their micro-environmental constituents is another contributing factor to their expansion. For instance, the interaction of stromal cell‐derived factor 1 (SDF‐1) with CXCR4, a G‐protein‐coupled receptor, considerably affects HSC proliferation, survival, and differentiation.^12^ Furthermore, direct cell-cell interactions mediated via various types of adhesion molecules play a crucial role in the fate of HSCs by affecting different mechanisms involved in self-renewal, differentiation, migration, quiescence, and apoptosis. Some of the most important adhesion molecules involved in HSC homing are integrins, selectins, N-Cadherin, notch receptors, CD44, esam1, cytohesin1, serum response factor (Srf), intercellular adhesion molecule 1 (ICAM-1), erythropoietin-producing hepatocellular (Eph) and ephrins as well as the SDF-1α/CXCR4 axis.^13^ Integrin-αvβ3 plays a fundamental role in the maintenance of HSCs through interaction with thrombopoietin, a crucial cytokine for the activation of dormant HSCs.^14^ Of note, ICAM-1 is essential for maintaining HSC quiescence and repopulation capacity in the niche, and in studies ICAM-1 deletion led to failure in the retention of HSCs in the bone marrow and changed the expression profile of stroma cell-derived factors.^15^

Three-dimensional culture systems are growing rapidly worldwide due to their ability to mimic tissue-like structures more efficiently compared to monolayer cultures particularly in cancer and stem cell research.^16^ A study conducted by Kumbhar et al. demonstrated that the inhabitability of UCB-MSCs was improved using 3D scaffold-based cultures through proper adhesion and proliferation.^17^ Furthermore, enhancement of the development and regulation of cellular signaling in stem cells using 3D cell platforms has also been reported.^18^ Multiple studies acknowledge that 3D microenvironments can promote cell viability and direct cell adhesion,^19^ proliferation,^20^ differentiation,^21^ and migration^22^ via the regulated presentation of mechanical and biochemical cues. Among the most widely used scaffolds, fibrin gel is superior in various aspects, such as high seeding efficiency and uniform cell distribution. Additionally, it can be harvested from the patient's own blood and used as an autologous scaffold excluding the potential risk of unintended reaction or infection.^23^

Previous research^24^ highlighted the favorable impact of fibrin on the increased expression of hematopoiesis-related genes in USSCs. In alignment with these findings, the present study focused on the expression of several adhesion molecule genes - ICAM-1, integrin subunit αv (ITGAV), and integrin subunit β3 (ITGB3) - in USSCs cultured on a 3D fibrin scaffold. This emphasis arises from the crucial role of direct cell-cell interactions in HSC expansion. Together, these studies provide a new perspective for further investigations into whether USSCs as stroma cells can effectively support HSC expansion in co-culture conditions on a 3D fibrin scaffold.

## Materials and methods

### Isolation and expansion of unrestricted somatic stem cells from umbilical cord blood

The procedures for the collection of human UCB units were performed after the informed consent of the mothers, in accordance with the Ethics Committee of the Tarbiat Modares University (IR.MODARES.REC.1399.026**)**. Experiments were performed with eight cord blood units**.** USSCs were isolated and cultivated according to the standardized protocol published by Kogler et al.^9^ The mononuclear cell fraction was first separated from UCB using a hydroxyethyl starch buffer (Santa Cruz Biotechnology, Santa Cruz, CA; sc-215159) followed by centrifugation (400 g for 25 min) on a Ficoll density gradient (Panbiotech, Germany; density 1.077 g/cm^3^; P04-60225). As a result, the solution inside the tube was divided into four distinct parts, serum, a layer of mononuclear cells, Ficoll, and red blood cells (RBCs). The separated mononuclear cells were plated out at 5-7×10^6^ cells/mL in T25 culture flasks with Dulbecco's modified Eagle's medium (DMEM) - low glucose (Gibco, 31600-083) supplemented with 30% fetal bovine serum (FBS) (Gibco; 10270106), 10^−7^ M dexamethasone (SigmaAldrich; D4902), 2 mM glutamine (Sigma; G8540), 100 U/mL streptomycin (Gibco; 122-15140), and 100 mg/mL penicillin (Gibco). The cells were incubated at 37°C with 5% CO_2_ in a fully humidified atmosphere. The culture medium was changed to DMEM supplemented with 10% FBS without dexamethasone after the appearance of adherent USSC colonies. The cells were split when confluency reached 80% by detaching the cells with 0.25% trypsin and re-plating them in a ratio of 1:3 under the previously described medium conditions.

### Monoclonal antibodies for the immunophenotyping of unrestricted somatic stem cells

The immunophenotype of the USSC cultures in the 3th passage (5 µL for 10^6^ cells) was investigated using the Attune NxT Flow cytometer. The following monoclonal antibodies were used: CD73-FITC (Biolegend, 344015), CD105-PE (Biolegend, USA; 323205), CD166-PE (Biolegend, USA; 343903), and CD45-FITC (Biolegend, USA; 304006).

### Differentiation of unrestricted somatic stem cells into adipocytes and osteoblasts

The differentiation protocol was based on the Kogler protocol.^9^ In the first stage, USSCs at the 3th passage were planted into six-well plates at a density of 5 x l0^3^ cells/well. For osteoblasts to be induced, after reaching 80% confluency, the culture medium was replaced with osteogenic induction medium supplemented with 10% FBS (Gibco), 10 mM β‐glycerol phosphate (Sigma Aldrich; 50020), 10^−7^ M dexamethasone (D2915), and 50 µg/mL ascorbic acid biphosphate (Sigma Aldrich; A8960). After 21 days of osteogenic stimulation, USSCs were fixed in 4% paraformaldehyde and stained with Alizarin Red (Sigma Aldrich; A5533) as an indication of osteoblast-typical calcification and functional competency of the differentiated cells. For induction of adipogenic differentiation, the same method was applied with the difference that the medium consisted of DMEM, 10% FBS, 250 nM dexamethasone, 60 nM insulin, 0.5 mM isobutyl‐ methylxanthine, and 0.2 mM indomethacin (all from Sigma‐Aldrich) in order to stimulate adipogenesis. Moreover, for the detection of lipid vacuoles, Oil Red O staining was used on the 21st day. The images were captured via an inverted microscope using a 200x magnification.

### Fibrin preparation

Utilizing a 3D scaffold for HSC expansion can mimic the bone marrow microenvironment, providing sufficient surface area for cell adhesion, as well as increased porosity to allow cell migration and nutrient exchange.^25^ In contrast, 2D expansion strategies significantly reduce HSC proliferation.^26^ Fabrication of fibrin gel was performed according to the method described by Soleimannejad et al.^27^ The fibrinogen solution was prepared by dissolving 1.5 mg of fibrinogen (Sigma Aldrich; F3879) in 0.5 mL DMEM and transferred to a 24-well culture dish. Next, 50 µL of FBS and 15 µL of a thrombin solution (120 U/mL in 1 M sodium buffer; Sigma, USA; 1.12374) were added to the fibrinogen solution (3 mg/mL). To allow for the formation of a 3D network structure, the plate was incubated at 37°C for 1 hour.

### Assessment of cell attachment

#### Scanning electron microscopy

To investigate the fibrin scaffold microstructure and cell-seeded fibrin gels, scanning electron microscopy (SEM) was used. After 12 hours of incubation, the specimen preparation for SEM analysis was according to the following procedure: in the first stage, the seeded cells on fibrin were fixed in 3% glutaraldehyde for 45 minutes at room temperature and rinsed twice in sterile phosphate-buffered saline (PBS). In the next step, the sample was kept at 4°C in PBS overnight and then dehydrated through a graded ethanol series solution (25%, 50%, 70%, 80%, 90%, and 100%). In the end, the prepared sample was dried and examined by SEM (XL30, Philips, Holland) under 1000x magnification.

#### 4′,6-diamidino-2-phenylindole (DAPI) staining

USSCs were fixed in 4% paraformaldehyde for 20 minutes at 4°C. After paraformaldehyde removal, cells were incubated with DAPI (Sigma) for 30 minutes at room temperature in the dark. After three washes using PBS, the DAPI-stained nuclei were observed using a fluorescence microscope (Nikon TE-2000) under 100x magnification.

### Assessment of cell viability by 3-(4,5-dimethylthiazol-2-yl)-2,5-diphenyltetrazolium bromide (MTT) assay

In Both the Control Group (without fibrin) and the Experimental Group (with fibrin), USSCs were seeded at a concentration of 1×10^5^ cells per well in a 48-well plate and were incubated (37°C and 5% CO_2_) for 1, 3, and 5 days. After the incubation period, 10 µL of the MTT labeling reagent (final concentration 0.5 mg/mL; 475989) was added to each well. Thereafter, the MTT solution was removed, and prepared DMSO was added to sufficiently dissolve the formazan crystals. In the final stage, the absorbance for each well was measured at 570 nm optical density using an enzyme-linked immunosorbent assay (ELISA) reader (BIOTEK, ELX800, Germany).

### Gene expression analysis of unrestricted somatic stem cells by quantitative polymerase chain reaction

Quantitative polymerase chain reaction (qPCR) was performed to evaluate the expression of the *ICAM, ITGAV*, and *ITGβ3* genes. USSCs in both Control and Experimental Groups were seeded at a density of 2×10^4^ cells per well into 24-well plates for 48 hours. Samples were then harvested and total RNAs were extracted using Trizol (Invitrogen: 15596018) according to the manufacturer's instructions. Subsequently, complementary DNA (cDNA) was synthesized using the cDNA Synthesis Kit (SMOBIO: RP1000) and qPCR was performed in an ABI StepOne PCR system (Applied Biosystems) over 40 cycles via SYBR Green Master Mix high ROX (Ampliqon, A325402). The primer sequences used for the qPCR are listed in (Table 1). The PCR products were run on 2% agarose gel electrophoresis and stained with ethidium bromide. *HPRT* was used as the housekeeping gene. Of note, all reactions were conducted in triplicate. Finally, relative gene expressions were analyzed using the 2^−ΔΔCt^ method.

**Table 1:** Primer sequences used for quantitative polymerase chain reaction.

Homo sapiens	Primer	Sequence
ICAM1	ICAM1/F	GAAGGTGTATGAACTGAGCAATG
	ICAM1/R	TGGCAGCGTAGGGTAAGG
ITGAV	ITGAV/F	TCCGAAACAATGAAGCCTTAG
	ITGAV/R	GCACACTGAAACGAAGACC
ITGB3	ITGB3/F	AACCGTTACTGCCGTGAC
	ITGB3/R	GGACACTCTGGCTCTTCTAC

### Statistical analysis

Data were analyzed using Graph Pad Prism® software version 9.0 (GraphPad Software, USA). All data are presented as means ± standard deviation (SD). For the MTT assay, data were analyzed using two-way repeated-measures ANOVA to evaluate the effect of fibrin on cell viability over the specified days. For qPCR, the normality of the data was assessed using the Shapiro-Wilk test, which confirmed that the data followed a Gaussian distribution. Statistical analysis was then performed using two-way repeated-measures ANOVA. Data are normalized to the *HPRT* gene.

## Results

### Characterization of unrestricted somatic stem cells

**Figure 1:**
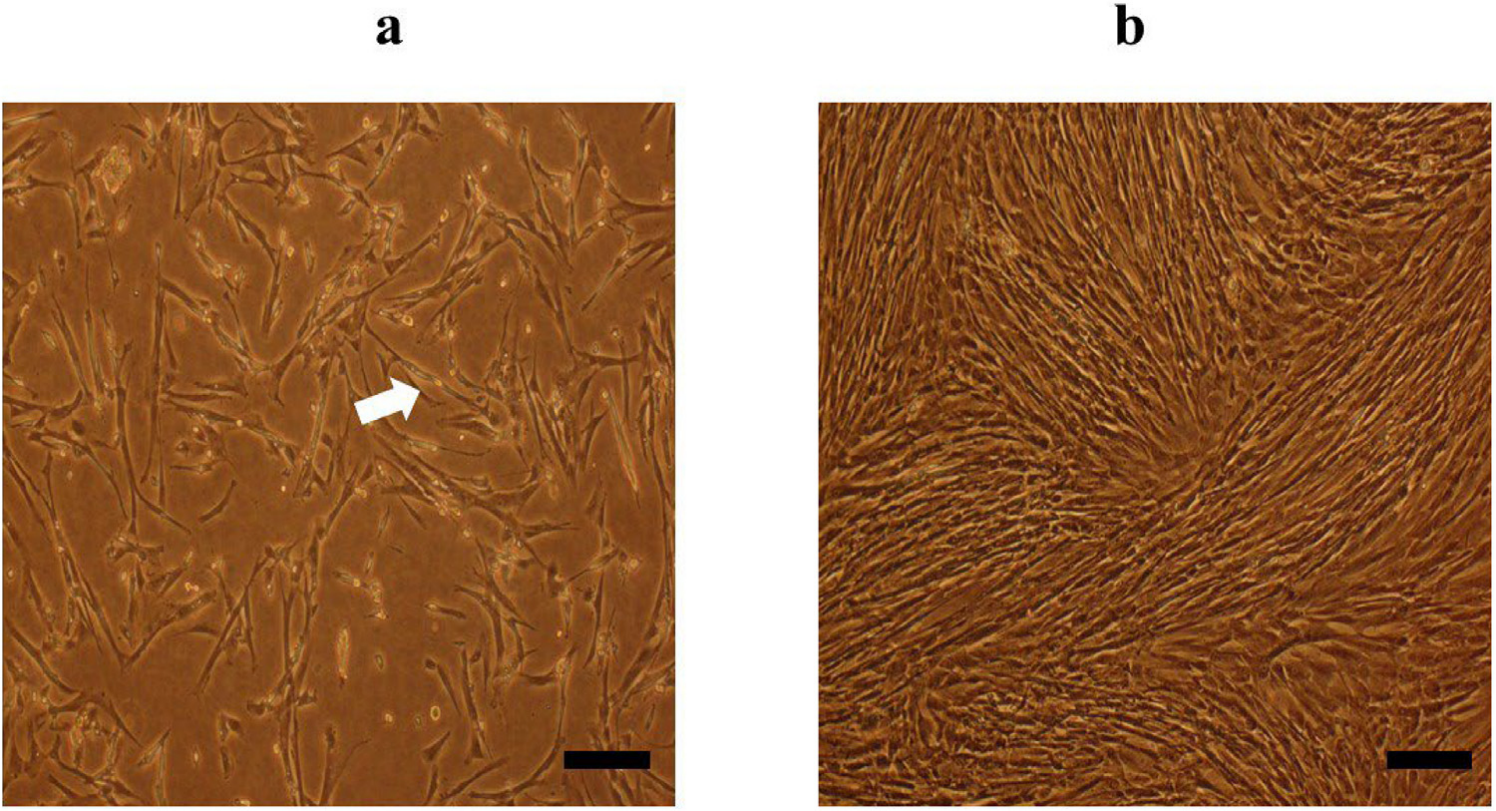
Morphological characteristics of unrestricted somatic stem cells (USSCs). (a) Spindle-shaped morphology of USSCs under an optical microscope on Day 3 with approximately 30% confluency. (b) USSCs at 80% confluency on Day 8. Both images (a) and (b) are from the Control Group (magnification 100x, scale bar = 50 µm).

**Figure 2:**
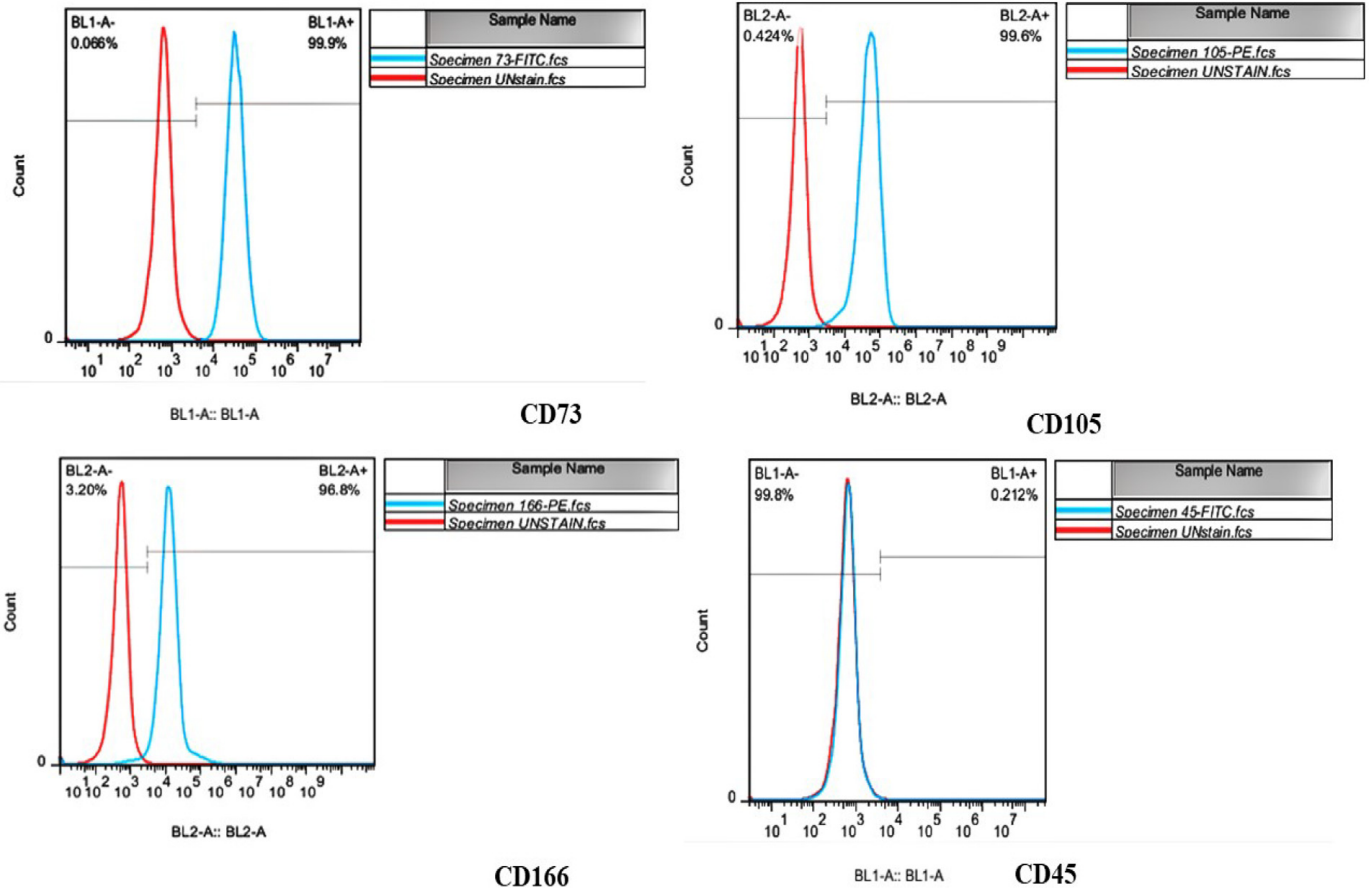
Immunophenotypes of unrestricted somatic stem cells (USSCs). USSCs were positive for CD73, CD105, and CD166 but negative for CD45.

**Figure 3:**
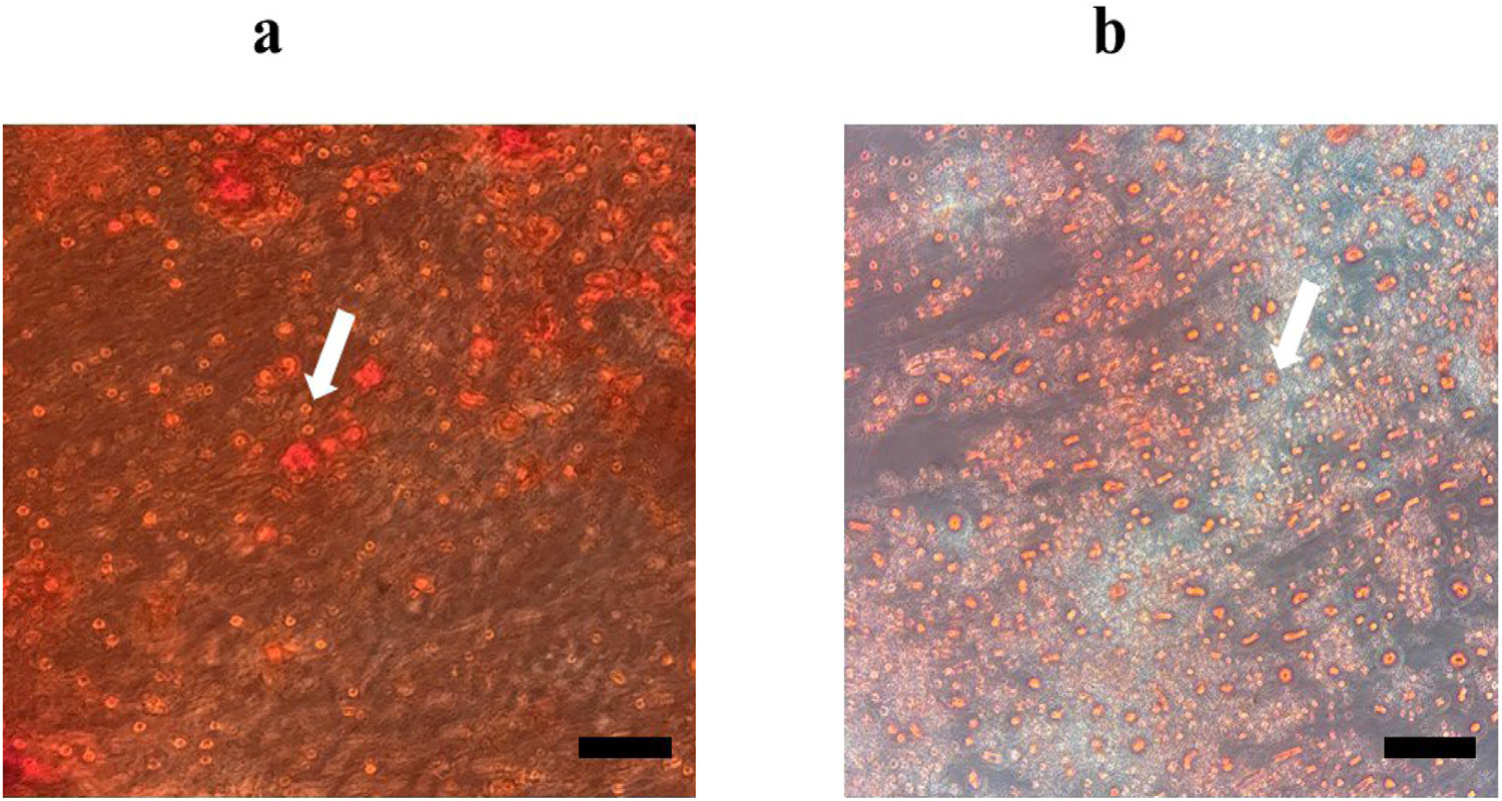
Multilineage differentiation potential of unrestricted somatic stem cells (USSCs). **(a)** After osteogenic induction, mineralized calcium nodules were detected using Alizarin Red staining (magnification 200x) **(b)** After adipogenic induction, lipid droplets were visualized by Oil Red O staining (magnification 200x).

In this study, USSCs were identified as adherent, spindle‐shaped cells as the arrows indicate in Figure 1a. This figure shows USSCs cultured on plastic tissue culture plates (Control Group) for three days, with confluency levels of approximately 30%. Figure 1b shows the same cells on Day 8 with confluency levels reaching around 80%. Isolated USSCs at the 3th passage were analyzed using flow cytometry to assess cell surface markers. The results show a cell surface expression profile of CD73 (97.9%), CD105 (97.2%), and CD166 (93.8%), with negativity (0.2%) for CD45 markers (Figure 2). The percentages provided came directly from the acquisition for total cells. USSCs have the potential to differentiate into both osteogenic and adipogenic lineages as confirmed by positive staining. Specifically Figure 3a demonstrates differentiation toward osteogenesis, as indicated by Alizarin Red staining for calcium deposits, while Figure 3b shows differentiation toward adipogenesis, highlighted by Oil Red O staining for lipid vacuole, which are indicated by arrows in Figure 3.

### Evaluation of cell attachment

**Figure 4:**
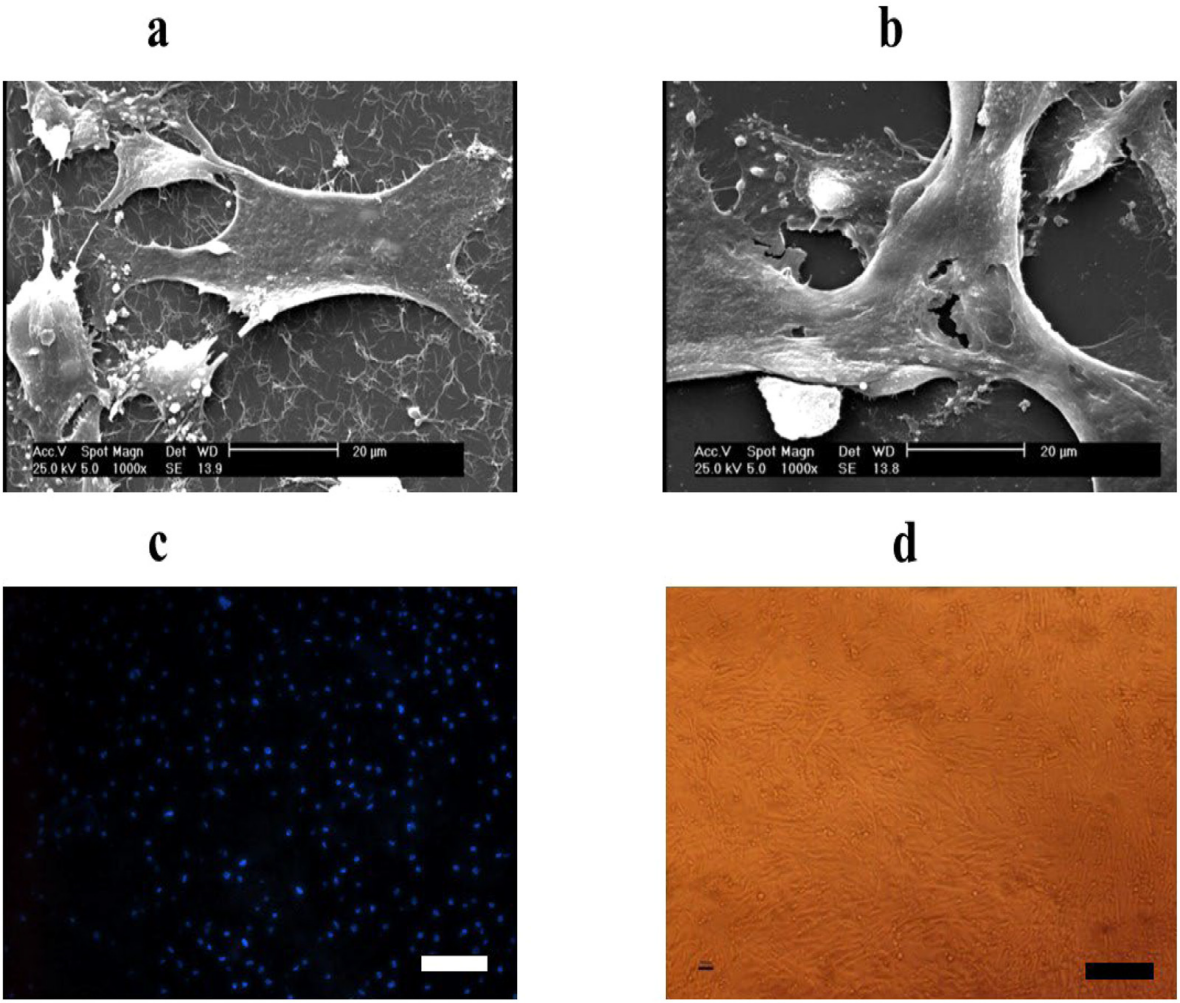
Cell attachment on fibrin. (a) Scanning electron microscopy (SEM) image of unrestricted somatic stem cells (USSCs) with fibrin. (b) SEM image of USSCs without fibrin. (c) Viable nuclei (blue) were shown using DAPI staining under a fluorescence microscopy. (d) Light microscopy image of USSCs attached to fibrin (scale bar = 50 µm).

As shown in Figure 4a, the images obtained by SEM analysis clearly show fibrin fibers with USSCs properly attached. In addition, Figure 4b demonstrates cells without fibrin networks. Fluorescence tracked DAPI-labeled USSCs cultured on the fibrin scaffold are shown in Figure 4c. The blue color corresponds to viable nuclei, with more blue spots indicating a higher number of viable cells attached to fibrin. The light microscopy image in Figure 4d also shows USSCs attached to fibrin, representing the Study Group. Altogether, these three sets of data confirm the adhesion of cells to fibrin.

### Evaluation of USSCs viability cultured on fibrin

**Figure 5:**
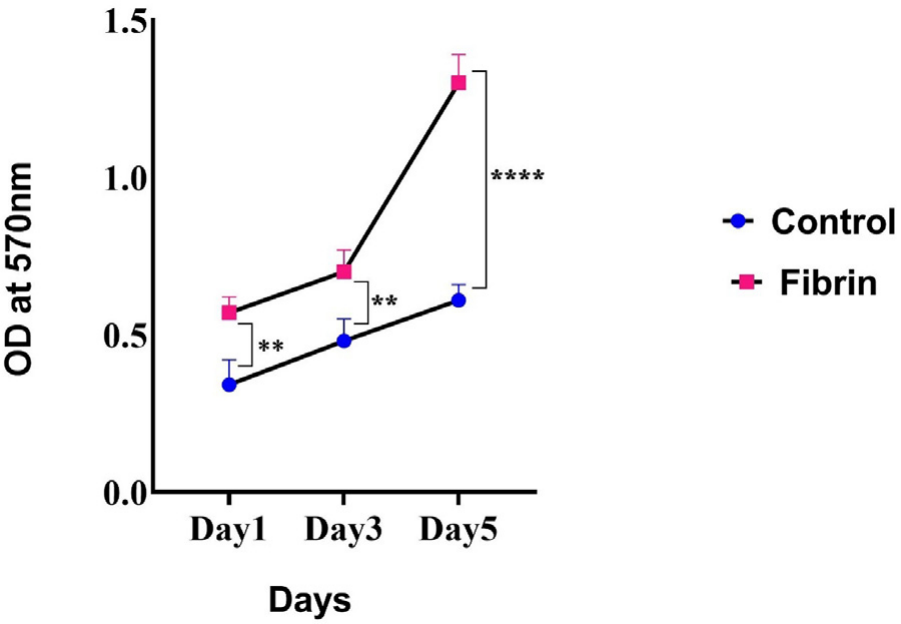
Effect of fibrin on unrestricted somatic stem cell (USSC) viability. The MTT assay result showed that fibrin has a positive effect on the proliferation of USSCs cultured on fibrin scaffolds at Day 1, 3, and 5 of continuous culture compared to those cultured on plastic tissue culture plates (Control Group). ^⁎⁎^p-value = 0.0051, ^⁎⁎^p-value = 0.0068 and ^⁎⁎⁎⁎^p-value < 0.0001 compared to control group.

Based on the results obtained from MTT assay, the proliferation of USSCs cultured on fibrin (Figure 5b) significantly increased compared to those cultured on plastic tissue culture plates (Control) on Day 1 (p-value = 0.0051), Day 3 (p-value = 0.0068), and Day 5 (p-value <0.0001).

### Expression of genes related to adhesion in unrestricted somatic stem cells cultured with fibrin

**Figure 6:**
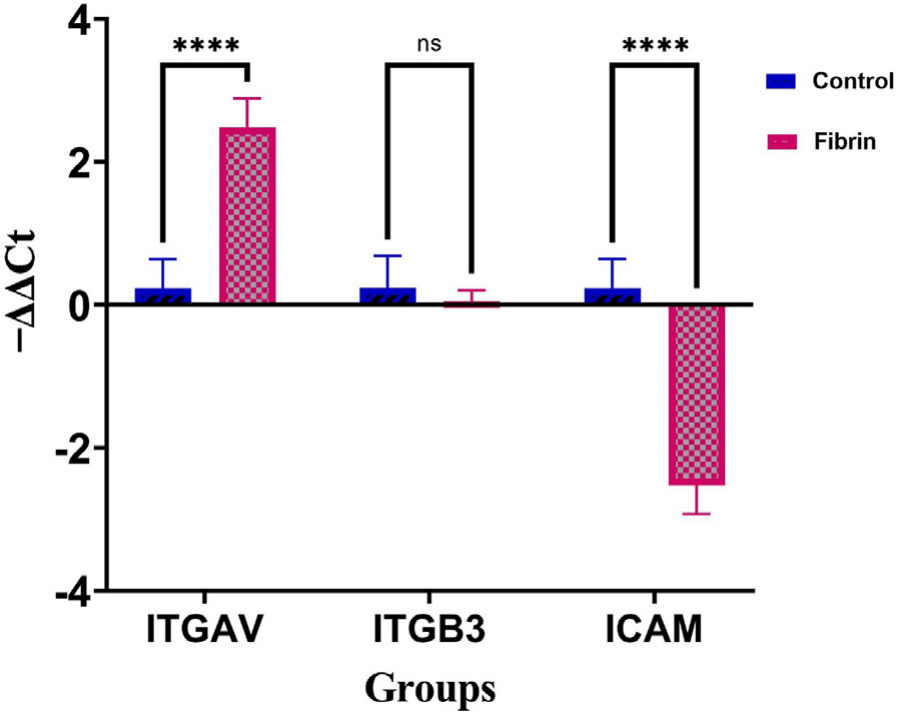
Effect of fibrin on expression levels of *ICAM, ITGAV*, and *ITGB3* in unrestricted somatic stem cells (USSCs). Quantitative polymerase chain reaction analysis revealed a significant increase in the mRNA expression levels of ITGAV in the fibrin group. Notably, fibrin did not exert an effect on the mRNA expression of ITGB3 in USSCs. The expression level of ICAM was significantly decreased compared to those cultured on plastic tissue culture plate. Data are presented as means ± standard deviation (SD). NS, not significant; ^⁎⁎⁎⁎^p-value < 0.0001 compared to Control Group (n = 3).

As shown in Figure 6, the expression of ITGAV was significantly higher in USSC cultured on fibrin compared to that cultured on plastic tissue culture plates (Control Group; p-value <0.0001). There was little difference in the expression of ITGB3 between the two groups (p-value = 0.2278). In contrast, the expression of ICAM-1 was downregulated in USSCs cultured on fibrin compared to those cultured on plastic tissue culture plates (p-value <0.0001). Information regarding the mean, SD and p-values for both groups is shown in Table S1.

## Discussion

UCB-HSCs have many beneficial aspects, including noninvasive collection, greater capacity of expansion, and remarkable tolerance in respect to HLA matching in transplantation thus making them a potential therapeutic candidate for hematological disorders. Nevertheless, their insufficient amount may lead to complications such as delayed engraftment.^12^ Therefore, finding a viable solution for the expansion of HSCs seems to be a promising area for future development. USSCs are potential candidates for stroma-driven *in vitro* expansion of CD34**^+^** cells from UCB to improve reconstitution and engraftment since they produce considerable amounts of functional hematopoiesis-supporting cytokines and are superior to MSCs in supporting the expansion of the UCB-HSCs.^8^ Moreover, in a recent study by Chan et al., USSCs promoted a significant enhancement of CD34**^+^** cell homing to the bone marrow and spleen.^28^ According to USSCs pluripotency and their expansion capacity into large quantities, they may serve as a global allogeneic stem cell source for various therapeutic options including transplantation, cellular therapy for tissue repair, and tissue regeneration.^9^ Therefore, *in vitro* expansion of USSCs may hold the key to tackling the issue of an inadequate number of HSCs for these treatments.

The HSC fate decision is controlled through direct cell-cell interactions - mediated via different types of adhesion molecules, - cell-ECM interactions - mediated mostly via integrins, - or through soluble mediators like cytokines.^13^ In other words, HSC adhesion to the substrate is assumed to be part of the natural process taking place in the HSC niche that regulates cell proliferation and differentiation.^29^ There is also evidence that cell adhesion is a known indicator of cell expansion.^30^^,^^31^ Integrins are one of the most important classes of the various types of adhesion molecules involved in the interaction of HSCs with their microenvironment.^13^ A study conducted in 2003, revealed that MSCs express various integrins, including ITGAV, and ITGB3 as well as ICAM-1, suggesting a possible *in vivo* role for these cells in both hematopoietic and immune function.^32^ Due to the ability of crosstalk between integrins and growth factor receptors through characteristic bidirectional signaling mechanisms, they can support cell proliferation and migration.^33^ Specifically, αvβ1 (VLA-5) along with α4β1 (VLA-4) and αLβ2 (LFA-1) play a crucial role in HSC adhesion to endothelial cells and their subsequent trans-endothelial migration toward the SDF-1α (CXCL12)-expressing stromal cells.^34^ Wierenga et al.^35^ reported a significant reduction in HSCs homing to bone marrow following the blocking of their αvβ1 integrins before transplantation.

In a study conducted on different types of 3D biomaterial scaffolds, fibrin achieved the highest overall growth rate of CD34^+^ HSCs, highest numbers of engraftment and multilineage differentiation, hence making it the most suitable option for *in vitro* expansion of UC-HSCs.^36^ Furthermore, another study revealed that seeding cytotoxic stem cells in fibrin scaffolds considerably elevated the initial retention and significantly prolonged the persistence and efficacy of the cells in the post-surgical brain cancer glioblastoma resection cavity.^37^ Moreover, fibrin also contributes to an increase in the expression of cytokines related to HSC proliferation, survival, and differentiation such as SCF and TPO.^24^

As expected, in this study the ITGAV expression was notably increased in USSCs cultured on fibrin compared to those cultured on plastic tissue culture plates which illustrates the positive impact of the fibrin scaffold on ITGAV levels. This result is in line with the findings of a previous study which highlighted the positive potential of fibrin on higher expressions of hematopoiesis genes such as SCF and TPO in USSCs cultured on fibrin.^24^ Previous studies show that integrin αIIbβ3 (CD41/CD61) probably plays a part in cell adhesion and cell surface-mediated signaling.^38^ Nonetheless, in contrast to its counterpart, USSCs cultured on fibrin displayed no significant changes in the *ITGBβ3* gene expression in this study. Surprisingly, there was a decreasing trend observed in *ICAM1* expression. Referring to previously published data,^24^ USSCs seeded on fibrin showed decreased levels of *Interleukin 6* (*IL-6*), which might be the underlying reason for the diminished *ICAM1* expression. Importantly, this reduction in mRNA level of *IL-6* was not attributable to the effect of fibrin and depended on atmospheric conditions (21% oxygen). Adjusting atmospheric conditions to 5% oxygen might result in the observation of positive effects on *IL-6* expression. Considering multiple studies indicating that *ICAM1* expression can be induced by *IL-6*, which can even promote its gene expression in endothelial cells and human osteosarcoma cells,^39^^,^^40^ the lower amounts of *IL-6* in USSCs may contribute to the reduction in *ICAM1*. It can be concluded that improving the expression status of both genes is achievable through alterations in oxygen conditions.

## Conclusions

This is the first time that the adhesive characteristics of USSCs on 3D fibrin scaffolds was studied. Taking together the findings of our previous and current study, in can be concluded that the fibrin scaffold demonstrates the potential to enhance the expression of various molecules such as SCF, TPO and ITGAV in USSCs. Therefore, in future studies, the co-culture of USSCs with HSCs on fibrin scaffolds looks promising due to the promoting effect of fibrin on the expressions of the aforementioned factors by USSCs. It is worth noting that according to previously mentioned studies, these adhesive and hematopoiesis factors play a fundamental role in HSC expansion and regulation in the bone marrow niche.

## Conflicts of interest

None.

## Appendix Supplementary materials

Image, application 1
