# Supplementary material for: Effect of fibrin on the expression of adhesion molecules (ICAM-1, ITGAV, and ITGB3) in unrestricted somatic stem cells
Source: Hematol Transfus Cell Ther. 2025 May 2;47(2):103827. doi: 10.1016/j.htct.2025.103827 (PMC12098137; doi:10.1016/j.htct.2025.103827)
Supplement: Supplementary file 1 [file mmc1.docx]

**Table S1:** Mean, standard deviation (SD) and p-values

|  | Control | Fibrin |
| --- | --- | --- |
| *ITGAV* |  | |
| Mean | 1.000 | 4.757 |
| SD | 0.02773 | 0.03297 |
| p-value | <0.0001 | |
| *ITGB3* |  | |
| Mean | 1.009 | 1.042 |
| SD | 0.1433 | 0.1120 |
| p-value | 0.2278 | |
| *ICAM* |  |  |
| Mean | 1.001 | 0.1480 |
| SD | 0.04160 | 0.002586 |
| p-value | <0.0001 | |
